# Imaging Approaches for the Study of Metabolism in Real Time Using Genetically Encoded Reporters

**DOI:** 10.3389/fcell.2021.725114

**Published:** 2022-01-18

**Authors:** Panagiotis Chandris, Christina C. Giannouli, George Panayotou

**Affiliations:** ^1^ Institute for Bioinnovation, Biomedical Sciences Research Center “Alexander Fleming”, Vari, Greece; ^2^ Biomedical Research Foundation Academy of Athens, Athens, Greece

**Keywords:** fluorescent sensor, fluorescence resonance energy transfer, Warburg effect, permuted fluorescent proteins, metabolism

## Abstract

Metabolism comprises of two axes in order to serve homeostasis: anabolism and catabolism. Both axes are interbranched with the so-called bioenergetics aspect of metabolism. There is a plethora of analytical biochemical methods to monitor metabolites and reactions in lysates, yet there is a rising need to monitor, quantify and elucidate in real time the spatiotemporal orchestration of complex biochemical reactions in living systems and furthermore to analyze the metabolic effect of chemical compounds that are destined for the clinic. The ongoing technological burst in the field of imaging creates opportunities to establish new tools that will allow investigators to monitor dynamics of biochemical reactions and kinetics of metabolites at a resolution that ranges from subcellular organelle to whole system for some key metabolites. This article provides a mini review of available toolkits to achieve this goal but also presents a perspective on the open space that can be exploited to develop novel methodologies that will merge classic biochemistry of metabolism with advanced imaging. In other words, a perspective of “watching metabolism in real time.”

## Introduction

The term metabolism is used to describe a vast field which actually comprises anything involving synthesis, recycling and breakdown of biological molecules in tight balance with the energy budget (production and waste). As the term is rather generic, it practically involves every metabolic reaction and metabolite trafficking inside a cell or systemic circulation and trafficking of metabolites between tissues and organs of multicellular organisms. Regarding pathophysiology, cancer research has been leading in the past few years a renaissance of the study of metabolism (Pavlova and Thompson, 2016; Altea-Manzano et al., 2020). Researchers though tend to classify diseases as “chronic” (e.g., irritable bowel syndrome, Crohn’s disease etc.), “degenerative” (for instance dementia) or “infectious.” One might be caught by surprise to find out that relief or aggravation or even therapeutic approaches for these diverse diseases might be metabolism dependent (Kaser et al., 2010). Further afield, even stemness has been proven to be tightly intertwined with the presence of certain metabolites (Carey et al., 2015; Schell et al., 2017; Tsogtbaatar et al., 2020). In addition, what we perceive as metabolic status, or even hormonal regulation of the body as a network, appears to be influenced -not to say coordinated- by the gut microbiome and its metabolites (Zhao S. et al., 2020). This repositioning of metabolism as a key aspect of current biomedical research propelled the advancement of sophisticated quantitative metabolic profiling methodologies, such as NMR and mass spectrometry, using hybrid approaches to analyze metabolites in solution (Chen et al., 2020) or even *in situ*, in tissues (Andersen et al., 2021).

In the last two decades there has been an explosion in the field of light microscopy, which resulted in the breaking of the diffraction barrier using super resolution approaches—both deterministic (SIM, STED) and probabilistic (PALM, STORM, GSDIM)—with variants or even hybrids (MINFLUX) of those optical methods (Sahl et al., 2017). Furthermore we had the implementation and constant expansion of diffraction–limited but much gentler and faster microscope systems, such as light sheet microscopes at different setups that allow accommodation of diverse biological entities, ranging from cells to whole organisms (Wan et al., 2019). Collectively, although super resolution has a constantly expanding irrefutable role in our understanding of how cells organize their subcellular entities (Baddeley and Bewersdorf, 2018), its role in dissecting highly dynamic phenomena in living systems is rather limited so far due to the increased phototoxicity by high light intensities, but also due to long acquisition times needed to paint the structural landscape, not only with increased resolution but also with increased precision. In parallel, confocal systems have become faster and more light-efficient and even wide-field microscopy has benefited substantially from highly sensitive and much faster cameras (sCMOS and EMCCDs). Overall, combining optical hardware improvement with the development of new genetically encoded fluorescent toolkits allows us to observe in a quantitative manner dynamic phenomena of metabolic nature, thus complementing metabolomics analysis by disruptive approaches, such as NMR and mass spectrometry.

In this mini review we will present a set of metabolite sensors targeted to distinct subcellular compartments. Further, we propose re-targeting of some sensors to monitor metabolites in different compartments along with suggestions for a new set of sensors for metabolites with emerging roles in biomedical research for which there are no available quantitative tools in intact biological systems.

## The Field

**FIGURE 1:**
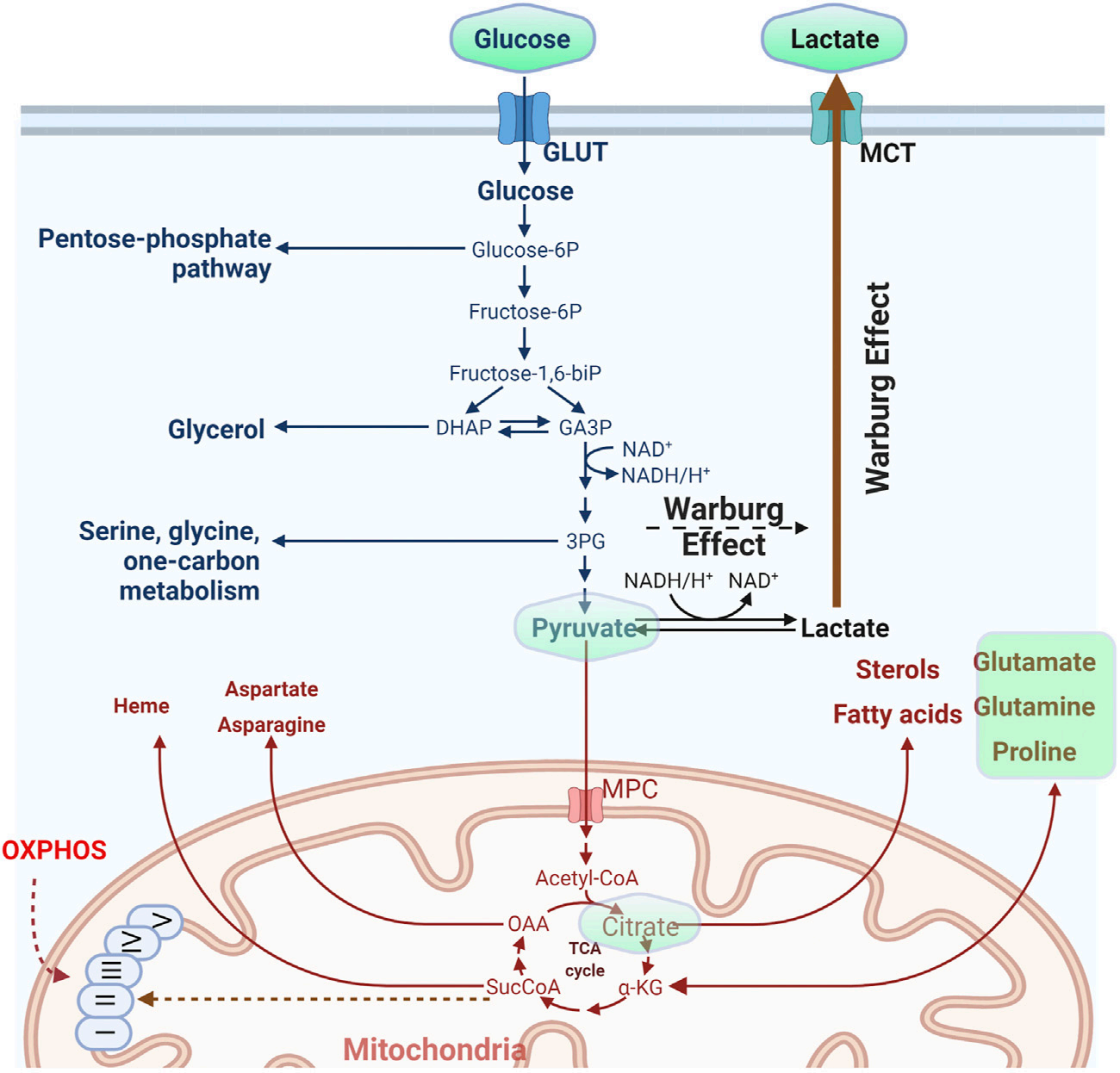
Outline of the basic metabolic pathway of glucose. The molecule is imported inside the cells and depending on the metabolic status might be used either for the synthesis of nitrogen bases through the pentose phosphate pathway or converted to trioses and from there to pyruvate. The last metabolite may either feed mitochondria or be converted to lactate and secreted in the extracellular medium. High rate of pyruvate conversion to lactate despite the presence of oxygen is called the “Warburg effect.” For some of the metabolites depicted in the figure, existing fluorescent reporters are described in the main text. Those metabolites are embedded in a light green frame. (Figure prepared by modifying a Biorender.com template).

The cell organizes its metabolism by compartmentalization. Sets of reactions take place in individual compartments and metabolites are exchanged either directly or indirectly by conversion to an intermediate metabolite that can pass a membrane barrier, (Lewis et al., 2014; Maddocks et al., 2014; Oeggl et al., 2018). It is also not uncommon that upon perturbation of a metabolic pathway cells will rewire their metabolic network to sustain viability and growth (Jiang et al., 2017) and this is always concerted with the balance of the redox potential of the cell (Hosios and Vander Heiden, 2018). Classic metabolic pathways include the uptake and metabolism of simple sugars such as glucose. The carbohydrate is imported into the cell with the action of transporters (Kayano et al., 1990; Chadt and Al-Hasani, 2020), gets phosphorylated and depending on the metabolic status of the cell, the hexose may be diverted to the pentose phosphate pathway to drive nucleotide synthesis or broken down to trioses. From that point on the cell may favor conversion to pyruvate and import it into mitochondria to support the Krebs cycle and oxidative phosphorylation (OXPHOS) along with energy production, or follow the anaerobic path and produce and secrete lactate (Figure 1). Glycolysis takes place in the cytosol while OXPHOS in the mitochondrial matrix. The pentose phosphate pathway occurs in the cytosol, yet the full path down to purine synthesis shuttles between cytosol and mitochondria.

In parallel to glucose metabolism, the cells may uptake other nutrients from the microenvironment including amino acids (Chantranupong et al., 2015; Efeyan et al., 2015). Glutamine for instance exerts a central role in metabolism, as it is regarded an “essential non-essential” amino acid. It is used in translation but it also serves to supply the cell with carbon and nitrogen. Glutamine may enter the cell and either get metabolized to glutamate in the cytosol or shunted to mitochondria where it is converted to glutamate and finally to a-ketoglutarate to feed the Krebs cycle (anaplerosis) (Figure 1). Alternatively, it can be diverted to non-essential amino acid (NEAA) synthesis *via* transamination reactions to support cell growth (Coloff et al., 2016). Elevated demand and metabolic rates for glutamine have been documented for many types of cancer (Zhang et al., 2017; Sniegowski et al., 2021) and evidence suggests that this nutrient’s uptake and metabolic reprogramming is directly connected to the action of oncogenes in cancer (Wise et al., 2008). As a result, mitochondrial glutaminase isoforms (the enzymes that hydrolyze glutamine in mitochondria) are emerging as important therapeutic targets. Glutamine metabolism is directly related to glutamate, which also plays an essential role in intracellular metabolism but furthermore functions extracellularly as a major neurotransmitter.

Although much attention has been given to mitochondria (for obvious reasons) regarding their role in metabolism, other organelles also hold a central role in metabolism, particularly for certain classes of metabolites. In light of the finding that a major orchestrator of metabolism, the target of rapamycin complex (TORC), shuttles on and off the lysosomes, this organelle is no longer considered simply a trash bin of the cell, but a hub of major recycling pathways, ranging from amino acid to sphingolipid metabolism (Sancak et al., 2008; Sancak et al., 2010; Betz and Hall, 2013; Sabatini, 2017; Wyant et al., 2017). Just to name a few more key players, peroxisomes are also key constituents for fatty acid synthesis and oxidation (Wanders et al., 2020), while the endoplasmic reticulum synthesizes phospholipids cooperatively with mitochondria (Jacquemyn et al., 2017).

Metabolism is a vast field and it is not the scope of this mini review to cover all aspects of metabolism–related sensors. We will mostly focus on the variety of sensor tools that have been developed to monitor key aspects of carbon and to some extent nitrogen balance, due to their importance and topical interest.

## The Design Rationale

### FRET and B-RET Based Sensors

Forster (or Fluorescent) Resonance Energy Transfer, is a physical process where energy migrates from an excited fluorophore to an adjacent one in a non-radiative manner. It is the result of long-range dipole-dipole coupling and it has a useful range between 10–100 Å (1–10 nm). The method has been extensively used to monitor protein-protein interactions, affinity and other dynamic parameters (Berney and Danuser, 2003; Bajar et al., 2016). FRET may occur between two different fluorophores (hetero-FRET) but also between two molecules of the same fluorophore (homo-FRET). Homo-FRET analysis is based on anisotropy measurements, requires specialized instrumentation and although it can be used for analyzing molecular dynamics and signaling events (Bader et al., 2009; Warren et al., 2015), it is not the method of choice when it comes to metabolite analysis.

**FIGURE 2:**
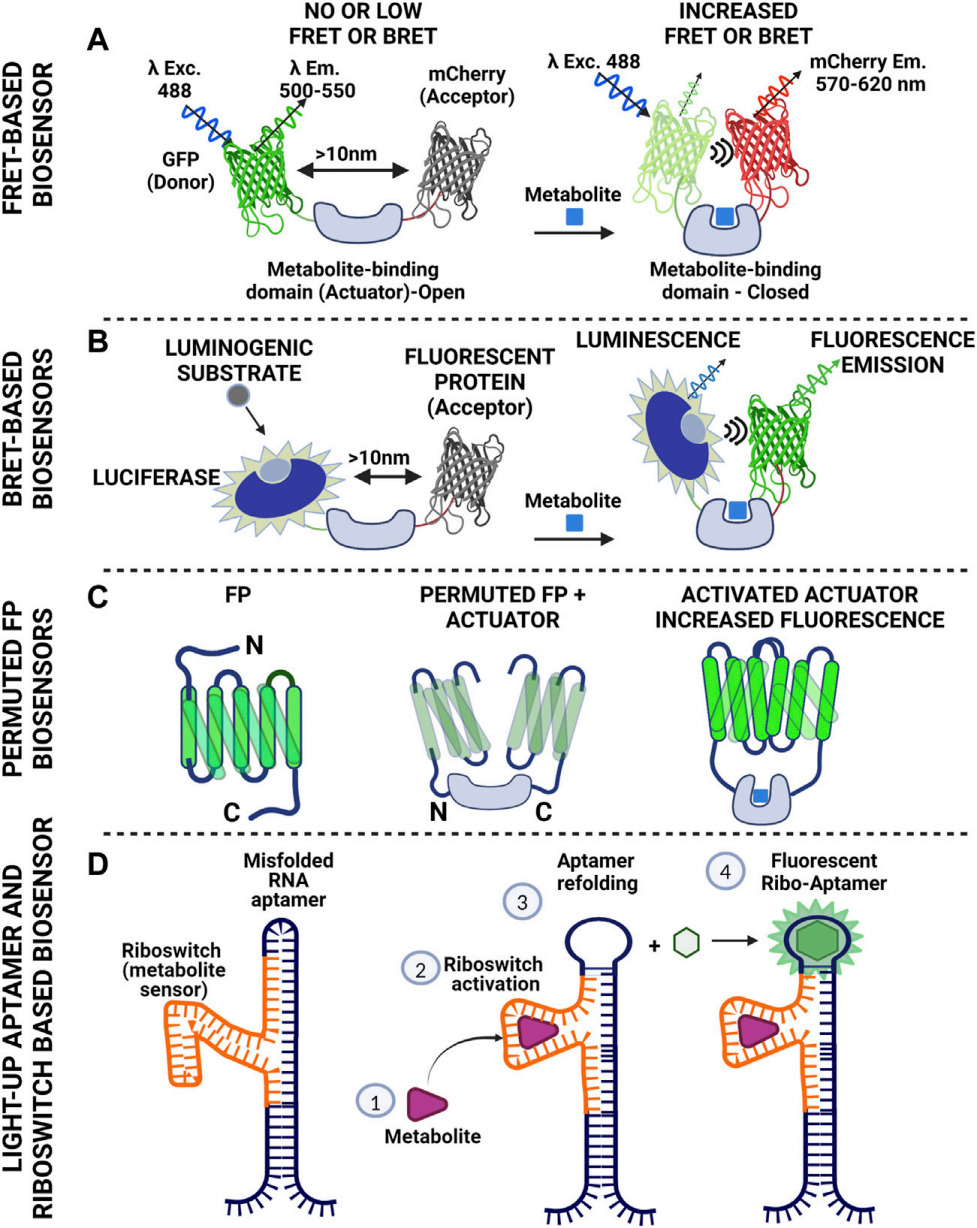
Schematics of basic tools used to construct biosensors for metabolite monitoring. **(A)** Cameleon-like Förster Resonance Energy Transfer design using fluorescent donor and acceptor with overlapping spectra. The fluorescent molecules are bridged with a protein domain that serves as specific metabolite sensor. Binding of the metabolite to the actual sensor (Actuator) triggers conformational changes that result in reduced distance between the two fluorescent proteins. Proximity facilitates energy transfer to the acceptor resulting in its excitation and subsequent photon emission. Of note, there is no direct photon transfer between the two molecules (non-radiative). This scheme gives positive read out signal (increased FRET). Reciprocally, the actuator might cause the fluorescent proteins to come in proximity in the absence of the metabolite and loosen its conformation upon metabolite binding. In this case the readout will be negative (reduced FRET). **(B)**. Bioluminescence Resonance Energy Transfer is an approach similar to FRET. The energy donor here is luciferase. There is no excitation light for the donor. In the presence of oxygen the enzyme catalyzes oxidation of luciferin (or other suitable substrate) and the reaction emits photons. The wavelength of this emission falls within the excitation spectrum of the acceptor. In a manner similar to FRET, energy is transferred to the acceptor causing the molecule to fluoresce. This scheme allows control of the timing of the recording, as luciferase will produce light when the substrate is supplied. At the same time though the readout will fade with time due to substrate consumption. **(C)** Single color biosensors, based on permutation of fluorescent proteins. One can shuffle fragments of a fluorescent protein (notice the rearrangement of the C- and N- termini of the protein after permutation) and introduce an actuator within the FP sequence compromising fluorescence. Metabolite binding by the actuator increases proximity of the FP domains thus increasing fluorescence intensity **(D)** RNA-based strategy for metabolite detection. The scheme includes a type of RNA (aptamer) that binds a fluorogenic substrate and becomes fluorescent (light-up aptamer). This feature though depends on the conformation of the aptamer. Inserting a fragment of RNA in the aptamer sequence that can identify a metabolite (riboswitch) can cause suboptimal folding of the aptamer and loss of fluorescence. Metabolite binding to the riboswitch causes refolding of the aptamer, which thus gains the ability to fluoresce upon substrate binding. The system has been used with success for imaging S-Adenosylmethionine in bacteria and lately in mammalian systems. (Figure prepared using biorender.com).

Regarding fluorescent reporters for monitoring metabolic activity, “cameleon” type systems are mostly used (Miyawaki et al., 1997; Lindenburg and Merkx, 2014). In this case, donor and acceptor (usually two fluorescent proteins with overlapping spectra) are fused together (thus securing the 1:1 ratio) and in between them, a protein domain is placed that binds the metabolite of interest. Upon binding of the metabolite, a resulting conformational change of the linker domain results in a modified distance between the two fluorophores, thus tuning FRET efficiency, read as change in fluorescence intensity (Figure 2A). Cameleon-type systems bypass the fluctuating ratio between separated donor and acceptor, yet normalization of FRET intensity should be done carefully, taking into account artefactual readouts attributed to cross-excitation and bleedthrough (Bajar et al., 2016). In addition, although overlapping spectra is the primary criterion for efficient FRET readouts, pairs of proteins with markedly different maturation times should be avoided (Shaner et al., 2005). An alternative readout regarding FRET pairs is through the affected lifetime of the fluorophore of the donor molecule (lifetime FRET, LT-FRET). In this case, instead of measuring the drop of intensity of the donor and the increased intensity in the acceptor channel, the statistical distribution of the time required for the fluorophore to emit photons after a pulsed excitation is measured (Datta et al., 2020). These measurements can be conducted in time (time-correlated single photon counting-TCSPC) or frequency domain (FD). The advantage of LT-FRET over intensity–based is that it is to a large extent (but not completely) independent of the concentration of the fluorophores. It should however be taken into account that lifetime FRET (LT-FRET) is a very sensitive technique that is prone to errors attributed to violation of the sampling rate (especially for time correlated single photon counting approaches-TCSPC).

B-RET (Bioluminescence resonance energy transfer) is a phenomenon similar to FRET, but in this case there is radiation coming from a luminescent molecule (luciferase activity in the presence of suitable substrate) and the photons emitted are in the excitation range of the acceptor. The readout is fluorescence that comes from the acceptor molecule and the useful distance is again within the 10 nm scale (Figure 2B). The method has been used to monitor protein-protein-interactions in living cells (Kobayashi et al., 2019; Kobayashi and Bouvier, 2021) but also for setting up biosensors.

### Single–Protein Based Fluorescent Reporters

Fluorescent proteins can have major parts of their sequence rearranged and yet retain their fluorescent properties. This type of sequence shuffling is called permutation. Permutations can be circular or non-circular depending on the way the protein segments are rearranged. Permutation takes place in nature by gene duplications and truncations or partial gene duplications and insertions (Vogel and Morea, 2006). Permuted fluorescent proteins though, exhibit higher sensitivity to environmental factors, such as ions and pH, and furthermore spectral shifts usually arise. Permutation of fluorescent proteins has been used in a variety of applications, ranging from monitoring of calcium fluctuations, to estimation of redox levels (Shui et al., 2011; Kostyuk et al., 2019; Kostyuk et al., 2020). The rationale behind shuffling a fluorescent protein is simple: one may insert a fragment of interest that will work as an actuator within the structure of a permuted fluorescent protein. This inserted fragment (or fragments) has affinity for a molecule of interest. Upon binding of the ligand, a conformational change will be reflected upon the intensity of the signal coming from the fluorescent protein and/or ratiometric changes on their spectra (Figure 2C). The great advantage is of course the single molecule approach that alleviates the burdens of FRET-based sensors, yet shuffling a protein sequence and inserting sensory domains is far from trivial. One though may set off by using published efficient permuted variants and implement the actuator of interest.

### RNA Aptamer-Based Sensors

RNA aptamers can bind to a fluorogenic molecule in a reversible manner and become fluorescent. They come in different “flavors,” acting as monomers (e.g., Spinach2, Broccoli, Mango), but also as dimers (Corn) (Warner et al., 2014; Warner et al., 2017). The fluorogenic substrate defines excitation and emission spectrum (Trachman et al., 2017a; Trachman et al., 2017b; Warner et al., 2017; Truong and Ferre-D'Amare, 2019). RNA aptamers may be used as single fluorescent reporters or as FRET pairs (Trachman et al., 2020). So how do we get to use them as sensors? The answer lies in the “RNA world” and more precisely in the combinatorial use of riboswitches with RNA aptamers. Riboswitches are sequences of RNA that are found in the 3′ UTR of mRNAs and fold in three dimensions, exhibiting affinity for certain small molecules and metabolites. In bacteria for example, riboswitches are used to sense availability of certain nutrients (Barrick and Breaker, 2007; Henkin, 2008), or even control their levels (Ruff et al., 2016; Sherlock et al., 2018). The challenge lies in the proper combination of riboswitch and RNA aptamer so that the riboswitch-induced conformational change upon ligand binding will produce a read–out of fluorescent intensity of the aptamer (Figure 2D) (Hallberg et al., 2017).

## The Toolkit

### Glucose Sensors

Glucose is one of the most fundamental metabolites, as it is used for energy production but also for the synthesis of metabolic intermediates ranging from complex carbohydrates to nucleotides that are used for RNA and DNA synthesis. Cells have specialized transporters to import the molecule (Navale and Paranjape, 2016). Glucose uptake has long been used in clinical practice for PET (Positron Emission Tomography) using for instance a radioactive isotope of FDG (fluorodeoxyglucose, a non-metabolizable analogue of the sugar) to trace tissues with supraphysiological metabolic activity, as is the case in cancer (Almuhaideb et al., 2011).

Given the central role of the metabolite, initial imaging efforts led to the creation of a CFP-YFP cameleon FRET based sensor with intervened bacterial glucose/galactose binding protein (GGBP) serving as the actuator of FRET response upon glucose fluctuations (Fehr et al., 2003). Improved versions of this system yielded a new set of reporters with dynamic range from micromolar to millimolar range (Takanaga et al., 2008; Bermejo et al., 2010). A new set of single fluorophore-based glucose sensors, the Green Glifons, have been raised by engineering previous versions that harbor the bacterial periplasmic glucose/galactose binding protein embedded into the Citrine variant of GFP core (Mita et al., 2019). These sensors cover a broad range of glucose concentrations, exhibiting a 5–8 fold increase in fluorescence intensity. They also show however significant affinity for galactose and this should be taken into account for those planning experiments using this carbohydrate to diminish ATP from glycolysis, increase lactate consumption and boost OXPHOS (Mot et al., 2016; Balsa et al., 2019).

A single–wavelength intensity–based glucose sensor with applicability in various systems, ranging from single cell to organismal applications, has also been reported (Keller et al., 2021). This sensor is based on permuted green fluorescent protein with a sensitivity range from 1 μM to 10 mM, practically spanning the largest part of concentration range for both *in vitro* and *in vivo* systems. The sensor exhibits significant response (up to 200% increase in fluorescence intensity) and flexible applicability, even allowing intravital imaging. Following similar strategies, sensors for mono (ribose) or di-saccharides (sucrose), have been developed and applied in non-mammalian systems or even in small animals such as *Drosophila* and *C. elegans* (Lager et al., 2006; Sadoine et al., 2020). Along with the above, a FLIM-based sensor has been reported (Diaz-Garcia et al., 2017; Diaz-Garcia et al., 2019) yielding a maximum lifetime change in the range of 0.38 ns, yet as with every FLIM measurement, special equipment is needed and read outs are not straightforward. A large number of photons is required for accurate assignment of the lifetime of a fluorophore, rendering the use of such sensors more cumbersome than anticipated.

### Pyruvate and Lactate Sensors

We have grouped these substances for two reasons: 1) the interconversion of one to the other through combinatorial heterotetramerization of lactate dehydrogenase (LDH) isoforms (Gerich et al., 2001; George Cahill, 2006; Valvona et al., 2016; Parks et al., 2020) ties up their biochemistry in such a manner that their relative ratio depicts physiological status in a more accurate way and 2) a set of developed sensors are ratiometric for the two molecules.

Pyruvate plays a crucial role for cellular metabolism, as it is imported in mitochondria and converted to acetyl-CoA to fuel the Krebs cycle, is used in transamination reactions, while also serving as a ROS scavenger, and in particular for H_2_O_2_ (Gray et al., 2014; Liu et al., 2018). Lactate is produced from pyruvate through the action of lactate dehydrogenase and is mostly released in the extracellular space. Systemically, the metabolite traffics through the bloodstream, reaches the liver and is converted back to glucose in a process known as gluconeogenesis. Conversion of pyruvate to lactate occurs at high rates when oxygen availability is limited (anaerobic conditions). It may however follow this route under normal oxygen conditions in a process called “aerobic glycolysis” or Warburg effect (Figure 1), named after the Nobel laurate Otto Warburg who described this phenomenon as a fingerprint of tumor physiology (Warburg, 1956). Although demonized because of its elevated levels in cancer, aerobic glycolysis also takes place in normal cells and tissues under conditions where a high proliferation rate is required, as it is also used to provide the cell with metabolic intermediates (Hume and Weidemann, 1979; Lunt and Vander Heiden, 2011; Liberti and Locasale, 2016; Prochownik and Wang, 2021).

Given that pyruvate is converted to lactate in the cytosol and then excreted, most efforts have centered on sensors that record cytosolic lactate or those that record lactate fluctuations in the extracellular milieu. An initial effort was with a mTFP1-Venus based FRET nanosensor harboring the lactate binding domain of the *E. coli* protein LldR, termed Laconic (LACtate Optical Nano Indicator from CECs), where upon lactate binding a decrease in FRET efficiency is recorded (San Martin et al., 2013). The sensor gave a low to modest response yet it was tested at a high concentration range (up to 10 mM); it was however pH sensitive, necessitating read out normalization. It is of particular importance for lactate sensors to validate their pH dependence since the extracellular concentration of lactate can be in the millimolar range (from 1 up to 20 mM or even higher in some cases) both *in vitro* and *in vivo* (Kuhr and Korf, 1988; Grist et al., 2018), pushing the limits of the sensor regarding its dynamic range but also its response in acidic environments. As a proof of principle, Barros and others used a nuclear–targeted version of this sensor in combination with the FRET–based glucose sensor FLII12Pglu700μδ6 (Takanaga et al., 2008) to monitor simultaneous glucose and lactate fluctuations in HEK cells after pharmacological inhibition of lactate export (Barros et al., 2013). The same sensor, along with the FRET–based pyruvate sensor Pyronic (San Martin et al., 2014) with a negative readout (reduced FRET upon pyruvate binding), has been applied to *in vivo* experiments using 2-photon and intravital imaging to monitor intracellular lactate levels in astrocytes and neurons after intravenous lactate administration. Adenoviral vectors carrying the sensors under the control of cell–type specific promoters, were injected in the primary somatosensory cortex of mice, with the data supporting a model of higher lactate uptake by neurons compared to astrocytes (Machler et al., 2016).

More recently, new lactate sensors have been developed based on a single permuted protein for lactate (termed Green Lindoblum) and pyruvate (termed Green Pegassos) (Harada et al., 2020), introducing part of the LldR protein (amino acids 86–260) for lactate or PdhR (1–260) for pyruvate between amino acids N145 and S146 of the GFP molecule that was used in the G-GECO calcium indicator (Zhao et al., 2011). These sensors exhibit increased specificity and good dynamic range of metabolite concentration (pyruvate saturation close to 1 mM and lactate exhibiting plateau close to 2 mM), with an increase in brightness that can reach up to 5-fold for Lindoblum at the highest concentration and 3-fold for Pegassos. Robert Campbell’s group recently reported a single protein fluorescent reporter named eLACCO1.1, created by inserting circularly permuted GFP (cpGFP) into the bacterial L-lactate binding protein TTHA0766 and improving the best candidate (out of 70 constructs) by directed evolution. The sensor appears to function as a dimer and requires Ca^2+^ concentration above 0.6 μM to function. The sensor exhibits a 5-fold increase in fluorescence in the presence of 10 mM lactate and was used for monitoring extracellular lactate in cells in culture but also in the brain (Nasu et al., 2021).

As mentioned before, pyruvate is a crucial metabolite that bridges carbohydrate metabolism to mitochondrial function and OXPHOS but even more, glycolysis and ROS scavenging. After the first FRET based reporter for pyruvate (San Martin et al., 2014), Bulusu and others created another one harboring the bacterial Pyruvate dehydrogenase complex repressor (PdhR) between mTurqoise and cpVenus 173 (Bulusu et al., 2017). Upon pyruvate binding the sensor exhibits negative read out (reduced FRET). Although a weak responder (maximum ΔR/R_0_ in the range of −15%), the construct was used to generate a transgenic mouse with ubiquitous expression of the reporter, called the PYRATES (PYRuvATE Sensor) mouse, attempting to link presomitic mesoderm (PSM) development with glycolytic activity. They used 2D cell culture models to record the pyruvate gradient within the expanding culture, finding a maximum reduction of FRET in the range of 16%. It should be noted though that the pyruvate concentration used to achieve such difference was supraphysiological (20 mM).

A BRET (Bioluminescence Resonance Energy Transfer) approach was undertaken to investigate the activity of the mitochondrial pyruvate carrier (MPC) (Bricker et al., 2012; Herzig et al., 2012) and its role in modulating the Warburg effect. The investigators tagged the MPC isoforms 1 and 2 with either luciferace (luc8) or Venus and titrated BRET efficiency in transfected cells under various conditions of exogenously added pyruvate in permeabilized cells (Compan et al., 2015). They named this sensor RESPYR and used it in HEK and INS-1 cells to investigate the Warburg effect using pharmacological approaches to control metabolite fluxes. A single protein fluorescent sensor (PyronicSF) was recently reported, using the same regulatory bacterial protein as in Pyronic, with greater dynamic range and sensitivity (almost 20-fold more sensitive than the initial FRET–based Pyronic sensor) and high selectivity (Arce-Molina et al., 2020). The investigators targeted the sensor to astrocyte mitochondria and used it to first estimate the concentration of pyruvate in mitochondria, reporting a concentration in the range of 30 μM (variable between cell types) and then extended their studies to monolayers of *Drosophila* perineurial glial cells, to investigate the role of the mitochondrial pyruvate carrier (MPC) in metabolism.

As mentioned at the beginning of this section, lactate and pyruvate have a tight relationship. Given that pyruvate stands at the crossroads of OXPHOS and glycolysis, it is advisable to measure *ratios* of lactate to pyruvate in every biochemical application. This is far from being trivial on the microscope stand. Recently though, a FRET based lactate to pyruvate sensor was reported from the same team that launched the Pyronic FRET sensor. The investigators used the *Bacillus subtillis* LutR transcriptional regulator that appears to bind pyruvate and lactate, and placed it between mTFP1 and cpVenus173. The Lapronic sensor (LActate/Pyruvate Ratio Optical Nano-Indicator from CECs) exhibits positive FRET values at increasing lactate concentration and negative readouts upon pyruvate increase (Galaz et al., 2020).

### Citrate Sensor

Citrate is a product of the Krebs cycle, which bridges carbohydrate with lipid metabolism (Figure 1) (Zhao et al., 2016; Haferkamp et al., 2020). Soon after the finding that citrate lyase activity actually links the Krebs cycle with histone acetylation and gene expression (Wellen et al., 2009), a set of FRET–based (CFP/Venus) sensors for the metabolite were reported, using part of the histidine kinase CitA from *Klebsiella pneumoniae* that harbors a citrate sensing domain (Ewald et al., 2011). The sensors were initially tested *in vitro* and in bacteria, but the system was later used to measure cytosolic citrate fluctuation in pancreatic beta-cells as a function of CDK1 signaling (Gregg et al., 2019). Honda and Kirimura (Honda and Kirimura, 2013) created a different set of fluorescent indicators for citrate based on circular permutation, yet utilizing the citrate binding domain of CitA. These sensors (CF98) exhibit a high dynamic range (from 0.1 to 50 mM), yet their response is pH dependent, and normalization should be carried out. Following the single fluorescent protein strategy and based on circular permutation, Robert Campbell’s group created a new set of citrate sensors by swapping the calmodulin (CaM)-RS20 domain from their previously reported Ca^2+^ indicator ncpGCaMP6s (Qian et al., 2019) with residues 4–133 of the CitAP domain of *Klebsiella pneumoniae*. Following directed evolution, they created two citrate sensors, one with increased signal upon citrate binding (Citron1) with a ΔF/Fmin ≈ 9 and one with inverse-response (reduced signal upon citrate binding), named Citroff1, with a ΔF/Fmin ≈ 18, compared to ΔF/Fmin ≈ 1.1 for the CF98 sensor (measurements done in isolated proteins in solution) (Zhao Y. et al., 2020).

### Glutamine and a-Ketoglutarate

Glutamine is the most abundant amino acid in the blood stream and serves a central role in metabolism (Yoo et al., 2020). It can be converted intracellularly to glutamate by the action of glutaminase and then glutamate may be converted to a-ketoglutarate or used for transamination reactions (Coloff et al., 2016). Despite the increasing interest in investigating glutamine metabolism and one of its main derivatives (a-ketoglutarate), the palette of genetically encoded sensors remains poor. Regarding glutamine, there is one FRET-based sensor (Gruenwald et al., 2012), with Teal (mTFP) and Venus proteins as the FRET pair (FLIPQ-TV sensors), using GlnH (periplasmic glutamine binding) as actuator. These sensors exhibit fair stability within the physiological pH range, but their readout response falls below 10%, with glutamine concentration in the nano- to micro-molar range. The response was negligible in most cell lines tested, despite the increased extracellular glutamine concentration (up to 5 mM). An alternative FRET–based sensor harbors the bacterial GlnBP as cameleon with GFP. GlnBP is incorporating the unnatural fluorescent amino acid L-(7-hydroxycoumarin-4-yl)ethylglycine (CouA) by replacing the N138 codon with an amber codon (TAG) and co-transforming the *E. coli* strain C321.ΔA with the pEvol-CouRS tRNA ligase. The fluorescent amino acid serves as a donor and GFP as the acceptor. The sensor was tested *in vitro* and exhibited a maximum 1.9-fold FRET ratio increase, with a response curve titrated for glutamine concentration from 0 to 50 μM. As mentioned above, this type of sensor requires coexpression of the appropriate tRNA ligase and is a system that has to overcome various technical and biological obstacles before it can be applied successfully (Elia, 2021).

Regarding a-ketoglutarate, there are no valid reporters for mammalian or invertebrate systems at present. There have been efforts though to generate FRET based reporters that harbor either the NifA transcriptional regulator from *Azotobacter*, which is involved in the nitrogen fixation process and has a ketoglutarate binding domain (GAF) (Zhang et al., 2013) or the monomeric PII or NtcA proteins, both of which are involved in nitrogen metabolism and carry ketoglutarate binding domains in-between CFP and YFP (Luddecke et al., 2017; Chen et al., 2018). These sensors have been tested *in vitro* and in bacteria only, usually have a modest negative readout (FRET reduction) and may require additional factors, such as ATP, in order to operate, thus hampering their potential as tools for *in vivo* imaging in higher eukaryotes.

### Glutamate

Glutamate has long been at the center of neuroscientists’ attention, given that this is the most abundant amino acid in the brain and has a central role as a neurotransmitter. A number of glutamate transporters (EEAT1-3) have been characterized that exhibit cell–type preference for their expression. Glutamate is crucial for balanced brain function, as low levels of the molecule have been linked to serious pathological conditions, such as dementia, schizophrenia, and epileptic seizures amongst others (Zhou and Danbolt, 2014; Volk et al., 2015). As such, the molecule has attracted scientific interest for the development of genetically encoded biosensors to monitor extracellular levels. In fact, all available sensors for this amino acid were developed for neuroscience research with no particular emphasis regarding the intracellular effect of glutamate on amino acid balance and bioenergetics. A major problem though in the redesigning of the sensors is that glutamate concentration is compartment–specific, varying by orders of magnitude (Featherstone, 2010).

A series of FRET–based sensors have been developed making use of the glutamate/aspartate binding protein Ybej from *E. coli* and ECFP/Venus (or Citrine) molecules (Okumoto et al., 2005; Tsien, 2005). A follow-up resulted in a version of a glutamate–sensing fluorescent reporter (GluSnFR) improved by a factor of 6.2 over the initial version, and was used to monitor glutamate release in cultured hippocampal neurons with the sensor being targeted to the plasma membrane (Hires et al., 2008). Subsequently, Looger’s lab created a single–protein fluorescent sensor based on permuted GFP intensity-based glutamate-sensing fluorescent reporter (iGluSnFR) again using the same YbeJ (or GltI periplasmic glutamate binding protein from bacteria as actual sensor), achieving a 6-fold increase in fluorescence upon addition of extracellular glutamate (Marvin et al., 2013). This was further improved by replacing eGFP with circularly permuted superfolded GFP, creating a series of SFiGluSnFR sensors that expand the concentration range and include chromatic variants. The iGluSnFR sensors were further improved [termed fast (iGluf) and ultrafast (iGluu)] so as to monitor the waves of glutamate release in synapses (Helassa et al., 2018), with speed of data recording in the range of 10 Hz. Robert Campbell’s group have also reported a set of single–protein glutamate sensors, introducing red variants from circularly permuted mApple (R-iGluSnFR1) in the palette, but also with different topology, including non-circular permuted variants (Wu et al., 2018). These sensors were used to monitor extracellular glutamate in HEK cells with affinities in the micromolar range.

### Sensors for Other Amino Acids

During the last few years, demand has increased for sensors monitoring the intracellular amino acid pool upon metabolic fluctuations. To this end, a set of single fluorophore histidine sensors was developed by embedding the bacterial periplasmic histidine sensing protein HisJ in the cpYFP, and exhibited a broad concentration range (up to 1 mM) and a response ranging from 2- to 5-fold (Hu et al., 2017). The sensors were also tested for measuring mitochondrial concentration of histidine, albeit in this case a careful pH titration had to be performed given the more alkaline environment of the mitochondrial matrix.

A sensor for L-methionine has recently been reported (Ko Wooseok, 2019). This tool is based on a methionine binding protein (MetQ) from *E. coli*, mutagenized to harbor four residues of the fluorescent unnatural amino acid CouA, which acts as a FRET donor. A fusion protein between the mutant MetQ and YFP results in a cameleon-type system that responds to the presence of methionine in the micromolar concentration range. The reporter was used to report the metabolite levels in FBS. Its capabilities though were demonstrated only *in vitro*, in a buffer system with an alkaline pH (9.0). Furthermore, the use of unnatural amino acids as FRET partners necessitates the use of wavelengths close to the UV range, which poses extra stress to cells.

A set of FRET–based (CFP/Venus) cysteine sensors has also been reported, based on the Cj0982 protein as the actual cysteine sensor, with a modest response at high cysteine concentrations (up to 20% increase in FRET efficiency upon binding of 1 mM cysteine) (Singh et al., 2020). Furthermore, Ameen and others created a lysine sensor with the lysine binding periplasmic protein (LAO) from *Salmonella* sandwiched between CFP and YFP (Ameen et al., 2016). The sensors exhibited a concentration range from micro- to milli-molar but their performance was tested in bacteria and yeast only. Recently a FRET–based sensor (CFP/YFP) was developed for BCAA (branched chain amino acids) that was named optical biosensor for leucine−isoleucine−valine (OLIVe) (Yoshida et al., 2019) using a leucine/isoleucine/valine-binding protein from *E. coli*. The sensor exhibited a good response in the presence of BCAAs, yet it also exhibited a modest response in the presence of cysteine or threonine and was affected by redox conditions. In general, setting up sensors for amino acids is not trivial given the common structural backbone. Most importantly, assessing the total pool of amino acids by cytoplasmic targeting of the sensor will probably give erroneous results, in particular under nutrient challenging conditions. The main hub of amino acid turnover and sensing is the lysosome, on the surface of which natural amino acid sensors are residing along with the main regulator of metabolism, mTORC1 (Rebsamen et al., 2015).

### RNA-Based Sensors for S-Adenosylmethionine

S-Adenosylmethionine (SAM) is a widely studied metabolite that is a universal donor for methylation reactions and is also directly linked to methionine metabolism and ATP levels, thus impacting physiology and the epigenetic landscape (Lu and Mato, 2012; Janke et al., 2015). Sensors for SAM exhibit a particular interest and were the first to use riboswitches from the bacterial world (Batey, 2011) and implement RNA aptamers with fluorogenic substrates for visualization. Fluorogenic compounds are non-fluorescent (or dim) when in solution. Upon binding to the aptamer they become fluorescent with their spectra resembling those of fluorescent proteins (Bouhedda et al., 2017). One of the main problems in using RNA aptamers and riboswitches to construct sensors, is their low levels in mammalian systems, either due to low expression or to misfolding and rapid degradation. Samie Jaffrey’s group from Cornell initially developed a sensor for SAM (Paige et al., 2012) using a stem sequence that acted as actuator along with the metabolite binding sequence, and the Spinach aptamer as a fluorescent reporter emitting in the green region upon binding of the DFHBI (3,5-difluoro-4-hydroxybenzylidene imidazolinone) fluorogenic substrate (Paige et al., 2011). This tool was initially used in bacteria, however, the same group recently introduced additional tools that have been implemented in mammalian cell culture systems. The first one is based on the SAM-III riboswitch and a Corn aptamer that forms dimers. Corn was engineered to be conditionally dimeric upon binding of S-Adenosylmethionine into its SAM-III riboswitch, causing binding of its fluorogenic substrate DFHO (3,5-difluoro-4-hydroxybenzylidene imidazolinone-2-oxime), which fluoresces in the yellow region (Kim and Jaffrey, 2019). The second tool involves the Red Broccoli aptamer, which is a monomer and along with a SAM riboswitch can glow into the red region upon binding to its substrate, OBI (3,5-difluoro-4-hydroxybenzylidene-imidazolinone-2-oxime-1-benzoimidazole), which is cell permeable and can be used in cell culture systems (Li et al., 2020). The same group has also implemented a system termed “Tornado,” based on circular RNAs. In this case, the RNA of interest is flanked by Twister ribozymes. Upon expression, ribozymes self-catalyze their cleavage, followed by ligation from the ubiquitously expressed RNA ligase RtcB, thus resulting in circularization of the RNA and increased stability. The system can harbor monomeric (Broccoli) but also dimeric aptamers (Corn) and was used with success in various cell lines to demonstrate detection of S-Adenosylmethionine (Litke and Jaffrey, 2019). The flexibility on the selection of the RNA aptamer and fluorogenic substrate also provides the benefit of multicolor imaging.

## Conclusion and Perspective

Being able to “watch biochemistry in real time” is essential in order to integrate knowledge from diverse areas of higher eukaryote metabolism and mammals in particular. Delving into complex biochemical pathways requires an interdisciplinary effort to develop novel tools that can address the spatiotemporal organization of biochemistry. In other words, where are things happening and in what order? Sensors for monitoring metabolites in real time have therefore attracted much attention and although technology has allowed us the expansion of available tools in particular for higher eukaryotes (see Table 1), further development is essential. Although metabolism is a huge field, if we were to pinpoint some aspects of “immediate need” we would first retarget some of the existing sensors in other subcellular compartments, such as mitochondria and the nucleus. This however is not as trivial as it sounds, since metabolite concentrations may change drastically, as for instance in the case of glutamate, which exists at low concentration extracellularly (micromolar range) but it jumps to the millimolar range intracellularly (Moussawi et al., 2011). Hence, not only one has to pick the right sensor but chances are that the tool will have to be rebuilt, taking into consideration rules and limitations that escort the designing of the reporter system (Deuschle et al., 2005; Fehr et al., 2005) (see also Table 2). In addition, and despite the fact that some metabolites, such as glutamine and a-ketoglutarate and the enzymes involved in their metabolism are targets of intensive research, we are still lacking toolkits for *in vivo* monitoring, at least in cell culture systems. Although fluorescent proteins have been the major tool to setup reporters, RNA-based reporters incorporating tools from the bacterial world are rapidly coming to the fore. One of the main challenges with the RNA probes has been their proper folding and stability, as well as the availability of cell permeable substrates. Recent implementation of a combinatorial use of ribozyme, riboswitch and aptamer resulting in circular RNA with significant stability is expected to broaden our palette of available tools for metabolite sensing. Last but not least is the question regarding “which microscope to use”? Is there any space for super resolution in metabolite sensing? Super resolution has provided significant insight regarding, for instance, mitochondria structure overall, with recent data from live imaging with Airyscan further supporting the notion that the organelle is not uniform (Wolf et al., 2019) and a metabolic gradient may appear within the same mitochondrion. It seems likely that super resolution modalities will be the optical tools of choice in cases where we need to monitor the interactions and nanoclustering of enzymes involved in metabolic pathways. On the other hand, confocal, wide field and variants of selective plane illumination (SPIM) microscopy, will be the primary choice for monitoring metabolites, in particular for small animal imaging. Finally, for those cases where we need to image long term or a wider field of view is required, light sheet microscopy which exhibits fast imaging with reduced phototoxicity looks set to become the standard.

**TABLE 1:** Listing of available biosensors for key metabolites.

Metabolite	Name	Sensor type	Biological system	Dynamic range	Reference
Glucose	FlipGlu	FRET	Cos-7 cells	Micromolar to millimolar	Fehr et al. (2003)
Glucose	Modified FlipGlu	FRET	HepG2 cells	Micromolar to millimolar	Takanaga et al. (2008)
Glucose	Green Glifons (various)	Single fluorescent protein	MIN pancreatic cells	Micromolar to millimolar	Mita et al. (2019)
Glucose	iGlucoSnFR	Circularly permuted GFP	Neuronal cells, *Drosophila*, Zebrafish	Micromolar to millimolar	Keller et al. (2021)
Glucose	iGlucoSnFR-TS	Fluorescence lifetime (FLIM)	Neuronal cells	Micromolar to millimolar	(Diaz-Garcia et al., 2017; Diaz-Garcia et al., 2019)
Sucrose/Trehalose/Glucose	FLIPsuc-90µ (various)	FRET	*In vitro only*	Micromolar to millimolar	(Lager et al., 2006; Sadoine et al., 2020)
Pyruvate	Green Pegassos	Single permuted fluorescent protein	HEK293, Hela cells	Micromolar (higher end) to millimolar	Harada et al. (2020)
Pyruvate	Pyronic	FRET	Astrocytes, HEK293, T98G glioma cells	Micromolar to millimolar	San Martin et al. (2014)
Pyruvate	PYRATES	FRET	*Ex vivo* Presomitic cell culture model	Micromolar to millimolar	Bulusu et al. (2017)
Lactate	LACONIC	FRET	Astrocytes, HEK293, T98G glioma cells	Micromolar to millimolar	San Martin et al. (2013)
Lactate	Green Lindoblum	Single permuted fluorescent protein	HEK293, Hela cells	Micromolar (higher end) to millimolar	Harada et al. (2020)
Lactate	eLACCO1.1	Circularly permuted GFP	T98G cells and *ex vivo* mouse brain tissue imaging	Micromolar to millimolar	Nasu et al. (2021)
Pyruvate	RESPYR	BRET	HEK293 cell culture	Micromolar (higher end) to millimolar	Compan et al. (2015)
Carrier activity					
Pyruvate	PyronicSF	Circularly permuted GFP	Mouse astrocyte cell culture and *Drosophila* dissected brain	Micromolar (lower end) to millimolar	Arce-Molina et al. (2020)
Lactate/Pyruvate ratio	Lapronic	FRET	HEK293 cell culture	Micromolar (from lower end) to millimolar (lower end)	Galaz et al. (2020)
Citrate	Cit96μ	FRET	Islet β-cells in culture	Micromolar (from lower end) to millimolar (lower end	Gregg et al. (2019)
Citrate	CF98	Circularly permuted fluorescent protein	*In vitro*	Millimolar	Honda and Kirimura, (2013)
Citrate	Citron and Citroff	Circularly permuted fluorescent protein	*In vitro* and Hela cells	Micromolar (lower end) to high millimolar	Zhao et al. (2020b)
Glutamine	FLIPQ-TV	FRET	Cos-7 cells	Nanomolar to micromolar	Gruenwald et al. (2012)
Glutamate	GluSnFR	FRET	HEK, Hela, Neuronal cells	Micromolar	Hires et al. (2008)
Glutamate	iGluSnFR	Permuted fluorescent protein	Mouse retina and neural cells and zebrafish	Micromolar	Marvin et al. (2013)
Glutamate	iGluf and iGluu	Circularly permuted GFP	HEK293 and neuronal cells	Micromolar	Helassa et al. (2018)
Glutamate	R-iGluSnFR1 and G-iGluSnFR	Circularly permuted fluorescent proteins	HEK293 and hippocampal neurons	Nanomolar to micromolar	Wu et al. (2018)
Histidine	HisJ	Circularly permuted YFP	Hela cells	Nanomolar to micromolar	Hu et al. (2017)
Methionine	YFPMetQ-R189CouA	FRET	*In vitro* (Serum)	Micromolar	Ko Wooseok, (2019)
Cysteine	Cys-FS	FRET	Yeast, HEK293	Micromolar	Singh et al. (2020)
Lysine	FLIPK	FRET	*In vitro,* Yeast	Micromolar	Ameen et al. (2016)
leucine−isoleucine−valine	OLIVe	FRET	Hela	Micromolar to millimolar	Yoshida et al. (2019)
S-Adenosyl methionine (SAM)	Corn-SAM	Corn RNA aptamer/SAM Riboswitch	HEK293T	Micromolar to millimolar	Kim and Jaffrey, (2019)
S-Adenosyl methionine (SAM)	Red Broccoli-SAM sensor	Broccoli RNA aptamer/SAM Riboswitch	HEK293	Micromolar to millimolar	Li et al. (2020)
S-Adenosyl methionine (SAM)	Tornado-Broccoli-SAM	Circularized RNA/Broccoli aptamer/SAM riboswitch	HEK293T	Micromolar to millimolar	Litke and Jaffrey, (2019)

The table includes mostly those biosensors that have been tested in higher eukaryotes. A brief description of the dynamic range is given. In many cases the reported biosensor includes a set of variants that cover the whole dynamic range with a complete description in the accompanying reference.

**TABLE 2:** Basic requirements and features for the construction and use of a metabolite sensor.

Guidelines for the use of a metabolic sensor	
Critical parameter	Important feature
Compartmentalization of metabolites	Concentration differences may exist for the same metabolite in different compartments (cytosol, mitochondria, nucleus, endoplasmic reticulum etc.).
Toolkit selection	Start by trying existing ones first! Permuted FP-based reporters are single molecule (read out as intensity difference) while FRET and BRET require 2 molecules. RNA aptamers may be used as single color readout (intensity) or as FRET pairs.
Sensitivity of the reporter	Always check if the dynamic range of the reporter falls within the physiological range of the system under study!
Specificity/selectivity of the reporter	One of the most essential features. Promiscuity (cross-reactivity with similar metabolites) must be kept at a minimum. A new reporter should first be tested *in vitro* regarding dynamic range and specificity.
Neutrality of the reporter	A reporter should be as “neutral” as possible (should not affect the metabolite levels, which is not always the case though!).
Reversibility of read out	It goes with affinity. The reporter should follow metabolite fluctuations with a minimum lag phase.
Environmental effect on the stability of the reporter	In most cases it is environment-dependent (pH, redox). Subcellular organelles exhibit major pH differences. Peroxisomes and mitochondrial matrix are on the highest end (pH ∼8–8.5). Lysosomes and secretory vesicles are on the lowest pH range (pH∼5.5 or lower), while Golgi is slightly acidic and cytosol and nucleus exhibit more neutral pH
Time scale of reporter maturation	This is of particular importance, especially when setting up “cameleon” type FRET reporters. Donor and acceptor should have comparable maturation lifetimes.
Photostability	Fluorescent proteins/tags prone to bleaching can give erroneous readouts especially for FRET based applications
Brightness	Permuted fluorescent proteins > FRET/BRET > RNA light-up aptamers (for mammalian systems).
Difficulty of read out/need for special equipment	Reporter tools are listed in descending order regarding “difficulty of read out”: Lifetime-FRET > Intensity FRET > BRET > RNA light-up aptamers > Permuted fluorescent proteins.
